# A Novel Tool for Gait Analysis: Validation Study of the Smart Insole PODOSmart^®^

**DOI:** 10.3390/s21175972

**Published:** 2021-09-06

**Authors:** Efthymios Ziagkas, Andreas Loukovitis, Dimitrios Xypolias Zekakos, Thomas Duc-Phu Chau, Alexandros Petrelis, George Grouios

**Affiliations:** 1 Department of Physical education and Sport Science, Aristotle University of Thessaloniki, 57001 Thessaloniki, Greece; eziagkas@phed.auth.gr (E.Z.); loukovit@phed.auth.gr (A.L.); 2 Digitsole SAS, 54000 Nancy, France; d.zekakos@group-epsilon.com (D.X.Z.); t.chau@digitsole.com (T.D.-P.C.); apetrelis@live.com (A.P.)

**Keywords:** smart insole, gait analysis, validation, 3D motion capturing, PODOSmart^®^, Vicon

## Abstract

The new smart insole PODOSmart^®^, is introduced as a new tool for gait analysis against high cost laboratory based equipment. PODOSmart^®^ system measures walking profile and gait variables in real life conditions. PODOSmart^®^ insoles consists of wireless sensors, can be fitted into any shoe and offer the ability to measure spatial, temporal, and kinematic gait parameters. The intelligent insoles feature several sensors that detect and capture foot movements and a microprocessor that calculates gait related biomechanical data. Gait analysis results are presented in PODOSmart^®^ platform. This study aims to present the characteristics of this tool and to validate it comparing with a stereophotogrammetry-based system. Validation was performed by gait analysis for eleven healthy individuals on a six-meters walkway using both PODOSmart^®^ and Vicon system. Intraclass correlation coefficients (ICC) were calculated for gait parameters. ICC for the validation ranged from 0.313 to 0.990 in gait parameters. The highest ICC was observed in cadence, circumduction, walking speed, stride length and stride duration. PODOSmart^®^ is a valid tool for gait analysis compared to the gold standard Vicon. As PODOSmart^®^, is a portable gait analysis tool with an affordable cost it can be a useful novel tool for gait analysis in healthy and pathological population.

## 1. Introduction

Gait analysis is the standard assessment tool used to quantify human locomotion, particularly by enhancing the understanding of kinematic and kinetic parameters during walking [1,2]. Gait analysis is now recognized as a valuable tool with many applications in various fields such as sport and health [3,4] and can be used for the assessment of medical conditions such as neurological or musculoskeletal diseases [5,6]. For example, in the field of rehabilitation, gait analysis can be used in different stages: for the initial assessment of the degree of pathology and guidance for the practitioner in his prescription; for the follow-up of rehabilitation and its impact on the patient; and for the final assessment to evaluate the benefit of the treatment [7]. For these purposes, accurate measurements of gait parameters are necessary [8]. Advances in technology over the years have led to the development of two-dimensional (2D) or three-dimensional (3D) gait analysis, which offer reliable and accurate measurements of human movement [5,7].

Essential components of the gait analysis involve accurate measurements of spatiotemporal and kinematic parameters. In order to measure these variables, different devices have been developed, including stereophotogrammetry-based systems and force plates [9]. Although these devices have gained popularity in the research field, they exhibit some shortcomings. Specifically, they are not portable, and their configuration limits the capture of a limited number of steps and in a laboratory setting [10,11]. Moreover, these devices are costly. According to Hailey and Tomie, it has been estimated that the average cost of a gait study is approximately $2000 [12]. In addition, gait laboratory equipment requires purchases that average $300,000 [5].

Emerging wearable technologies, capable of measuring gait related parameters have been recently applied to gait research, opening up a novel approach to evaluate such parameters. The use of body-worn devices has been recently proposed to address the need for monitoring and quantifying motion. Among these devices, the smart insoles are the most common and state-of-the-art technologies. Sensors that are attached to the insoles can analyse gait on real time. A recent review concluded that inertial measurement units (IMU) are the most widely used sensors while insole pressure sensors are used in validation studies [13].

The sensor-based insoles provide measurements about spatial and temporal gait parameters without the constraints associated with traditional devices. For example, they are capable of providing measurements without wires. In addition, they collect data in both laboratory and field settings. This implies that the flexibility of the insole systems allows for the easy and quick application, analysis and feedback in the context of daily life activities. Furthermore, it is evident that the smart insoles can be easily applicated in diverse contexts such as sports performance analysis and prevention of injuries [14]. Based on the above the establishment of accurate and efficient wearable insoles is of great importance.

There have been several research efforts to develop and validate insole systems. One of the most commonly used sensor-based insole systems, the Pedar system, was found to be repeatable and valid [15,16]. According to the researchers, these insoles are capable of long-term measurements of the vertical force when corrected for offset drift. The study of Braun et al., in 2015 examined validity and reliability of “OpenGo” against pressure plate in 15 variables concerning kinetic and spatiotemporal gait parameters. Their results showed that intra-class correlation for the validation was >0.796 for temporospatial and kinetic gait parameters. As regards reliability, intraclass correlation was >0.994 [17]. In 2016, Truong et al. developed the insoles “Footlogger” in order to estimate walking distance with respect to the stride counting and concluded that this method provides a reliable result [18]. Later, Stöggl and Martiner (2017) validated the wireless sensor insole system “OpenGo” which was launched by Moticon GmbH, Germany. Researchers concluded that these insoles can be used to measure temporal, force and balance parameters during different types of motion such as walking or jumping [19]. Similarly, previous studies confirmed the validity of other insole systems such as “wi-GAT” [20], “eSHOE” [21], Medilogic [2] and plantar pressure redistribution insoles [22]. It should be noted that these studies have introduced sensor insoles that are suitable for different age groups or populations. For example, recently, Duong et al. (2020) presented the PediaSole targeted at pediatric population [23]. In 2019, the study of Burns et al. showed that a wireless shoe insole was valid for ground reaction force measurement in hopping, walking, and running [24]. In recent bibliography related to the validation of insole devices for gait analysis, previous studies examined the validity for only a few variables such as step counting and cadence [24,25], vertical force [15,16], or a small number of spatiotemporal gait characteristics [2,17,20,22].

To overcome these limitations in gait analysis using insole systems, Digitsole SAS has developed and commercialized the PODOSmart^®^ insoles. Unlike other sensor-based insole systems, PODOSmart^®^ has been designed to be compact, with high battery and memory storage regarding the size of the hardware and its location. These new low-cost insoles (around $2000 for the complete package) consist of completely wireless sensors with integrated internal storage. They can fit into any shoe and are able to measure 20 gait variables including spatial, temporal, and kinematic gait parameters in healthy individuals and specific populations, such as orthopedic and neurologic patients. Innovatively, they can be used for collecting data during different gait tasks. Their portability makes the analysis easier in various contexts or conditions. In addition, they allow an extensive range of motion to be achieved and can be used for measurements over more extended periods of activity. Hence, PODOSmart^®^ appears promising for applications in research and in various other fields such as clinical settings and sports fields under ecological environment. PODOSmart^®^ is using Bluetooth low energy and the insoles can be recycled. It also allows the healthcare professionals to send the report directly to the patient by email in PDF, so encouraging to avoid printed versions.

This study aims to introduce the PODOSmart insoles and to validate them using a stereophotogrammetry-based system, the gold standard Vicon system (OxfordMetrics, Oxford, UK). More specifically, to quantitatively assess the accuracy of the gait parameters provided by PODOSmart^®^ (Digitsole SAS).

## 2. Materials and Methods

### 2.1. Sample

Age and gender is already known to have a significant effect on human gait [26]. More specifically females present lower walking speed and shorter stride length than males [26]. Furthermore, research data have shown a significant effect of gender on kinematic features of gait [27]. The sample of the validation study consisted of 11 healthy male adults without diagnosed gait abnormalities. Their age ranged from 20 to 49 years (mean 33.50 ± 8.23 years). Their mean height was 173.40 ± 5.59 cm (16 cm range), and their mean weight was 74.73 ± 6.51 kg (20 kg range). Participants were informed about the objectives of the study and provided written informed consent to participate in the study. Their participation in the study was voluntary, and they were briefed about their right to withdraw from the study without any prior notice. In addition, participants were reassured about the confidentiality of their data and encouraged to ask questions regarding the process at any time. Ethics approval for this study was granted by the respective committee of the Department of Physical Education and Sport Science at the Aristotle University of Thessaloniki.

### 2.2. Instruments

#### 2.2.1. PODOSmart^®^ Insoles

**Figure 1:**
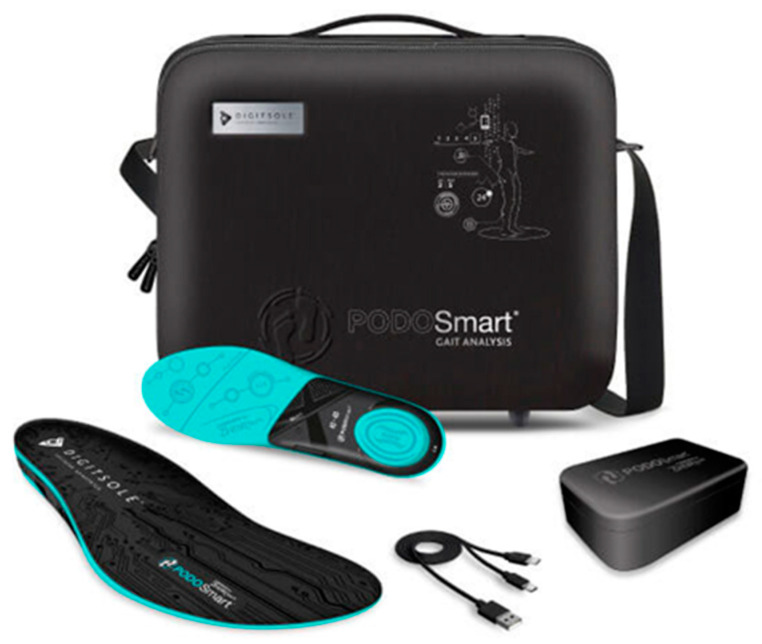
PODOSmart^®^ insole device.

**Figure 2:**
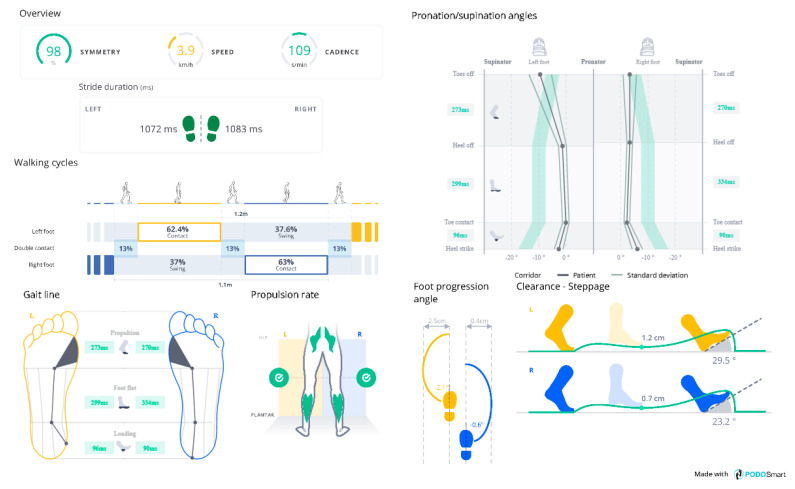
The graphical interface of PODOSmart^®^ gait analysis report.

PODOSmart^®^ system (Digitsole SAS, Nancy, France) consists of pairs of insoles (weighted a mere 66 g each and comes in six different sizes from 36 to 47 in FR shoe size-chart (5.5 to 12.5 US shoe size-chart, 3 to 11.5 UK shoe size-chart) to fit all adult populations—Figure 1) connected to Bluetooth connection box. The PODOSmart^®^ smart insole is rechargeable via USB for continuous 33 h with active use. Although PODOSmart^®^ smart insoles are capable of long-lasting recordings, PODOSmart^®^ artificial intelligence algorithms allow short-term recordings lasting less than a minute. PODOSmart^®^ device allows to measuring walking and running parameters of users, in real-life conditions. Each PODOSmart^®^ insole has an inertial platform that records each foot’s walking steps, running strides, and orientations in space with sampling frequency of 208 Hz for walk analysis. The Bluetooth connection box retrieves the collected data by the smart insoles. Then, those data are processed by proprietary artificial intelligence algorithms to calculate the spatiotemporal, kinematic, and biomechanical parameters (Table 1) [13,28,29,30], displayed in a graphical interface and processed into clinically usable data (Figure 2).

#### 2.2.2. Vicon Motion Capture Device

The Vicon-T40 3D (Vicon MX, Oxford Metrics, Oxford, UK) motion recording system consists of: (a) 10 infrared cameras with a high-resolution scale of 1 Mp and a shooting frequency of 250 fps (Bonita model 3), (b) the central unit (named by the company “Vicon Motion Systems Ltd.” “Lock Sync box”) which connects, organizes and synchronizes the communication of the 10 cameras and (c) a computer on which the software of Vicon Nexus version 1.7.6 (OxfordMetrics, Oxford, UK) is installed (d) and the central unit connected to the system. The special “wand” with five reflectors of the company “Vicon Motion Systems Ltd.” is used to calibrate the system. Spherical plastic reflectors with a diameter of 9.5 mm are used to calculate the kinematic characteristics. The segment and joint angular kinematics calculations are performed in NEXUS software (1.7.6) based on the 3D coordinates of the reflective markers placed on selected anatomical landmarks on the body [31]. The capture volume of the Vicon system was set to approximately 6 m × 6 m × 3 m.

### 2.3. Experimental Protocol

**Figure 3:**
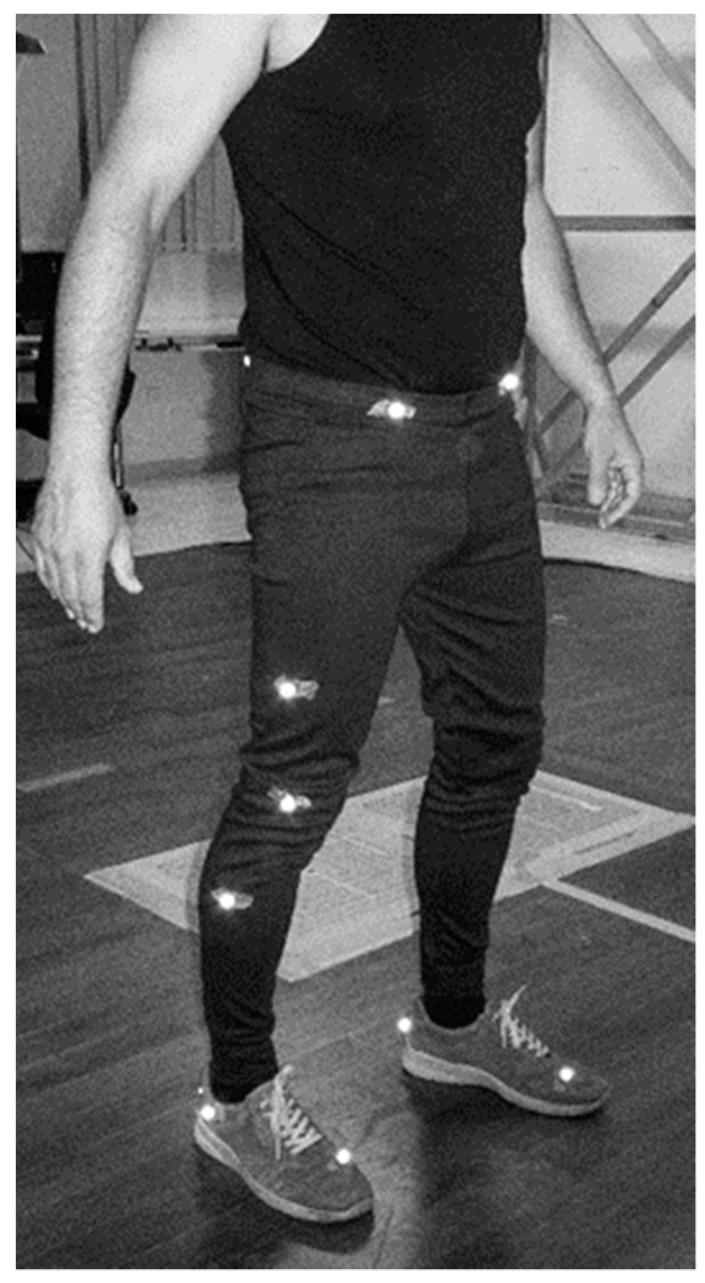
Vicon markers set configuration on subject.

Gait analyses were conducted in the “Laboratory of Motor Behavior and Adapted Physical Activity” at the Department of Physical Education and Sport Science of the Aristotle University of Thessaloniki. Participants wore their preferred walking shoes, tight t-shirt, and tights or tight shorts. The PODOSmart^®^ insoles fitted into participant’s shoes and 16 reflective markers of the Vicon system were bilaterally attached to specific anatomic points of the participants’ lower limbs according to the lower body plug-in-gait model enabling three-dimensional analysis of lower body movements during gait (Figure 3) [32]. A 6 m walkway was used for the analysis. The Vicon system cameras were positioned around the walkway and were calibrated to the walkway using standardized protocols recommended by the manufacturer at the beginning of each capturing session.

Participants were asked to walk at their preferred walking speed. In particular, participants began to accelerate to their self-selected comfortable speed at the first two meters of the walkway, and they decelerate at the last two meters to stop at the end of the walkway. Although all the 6-m walkway was captured using the Vicon system, only the middle four steps (two gait cycles) on the walkway were used for the analysis. Practice trials were performed to allow familiarization with the equipment and the experimental procedure.

### 2.4. Gait Analysis

Data from both devices were collected and stored during the execution of six steps for each participant (acceleration and deceleration steps were deleted from the analysis). Mokka (Motion Kinematic and Kinetic Analyzer, version 0.6.2), an open-source and cross-platform software to easily analyze biomechanical data [33], was used to read the .c3d files and the lower body plug-in-gait model outputs as exported by Vicon Nexus [31], visualize them in a 3D space, and annotate the four-step events for each walking cycle (heel strike (HS), flat foot in (FFI), flat foot out (FFO) and toe-off (TO)). Data from Vicon were then exported in a .csv file, including the timestamps of annotated events and data acquisition (i.e., the left and right 3D coordinates of the heel, toe, and ankle).

Afterward, the following gait parameters were calculated separately for the left and the right foot:

- Stride length (m): distance of foot displacement between two consecutive steps on the same side.
- Stride duration (ms): duration of foot displacement between two consecutive steps on the same side.
- Stance time (%): Duration of contact between the foot and the ground during one stride cycle. It is normalized to be expressed as a percentage of the stride duration.
- Swing time (%): Duration with no contact between the foot and the ground during one stride cycle. It is normalized to be expressed as a percentage of the stride duration.
- Foot progression angle (deg): angle between the orientation of the foot and the user’s trajectory.
- Circumduction
- Clearance
- Loading time (ms)
- Flat foot time (ms)
- Heel strike angle (°)
- Propulsion rate (%)
- Propulsion time (ms)
- Supination angle at heel off (°)
- Supination angle at heel strike (°)
- Supination angle at toe off (°)
- Supination angle at toe strike (°)

The three following gait parameters were calculated from data of both feet:

- Cadence: number of steps per minute; it is the sum of both the feet.
- Speed (km/h): average walking speed of the user overall steps.
- Double contact (%): duration of simultaneous contact between both feet and the ground. It is normalized to be expressed as a percentage of the stride duration.

Finally, the variable symmetry expresses the congruence between the values obtained for the left and right foot [13,28,29,30].

### 2.5. Statistical Analysis

Statistical analysis was performed using SPSS statistical software (version 25). From each walking trial, six steps were analyzed. Mean, and standard deviation of all steps of each subject were used for the comparison between the two devices. Gait data were screened for normality with the Kolmogorov–Smirnov Test. The validity of the smart insole was then explored for all metrics using the intraclass correlation coefficient (ICC) [34]. The appropriate forms of the ICC were the ICC intra-rater reliability, absolute agreement, as reported by Shrout and Fleiss [35]. This ICC illustrated the absolute agreement for multiple measurements and was generally considered as being either poor, moderate, good, and excellent reliability for values less than 0.5, between 0.5 and 0.75, between 0.75 and 0.9, and greater than 0.90, respectively [36]. The *p*-value was set at the level of 0.05.

## 3. Results

The ICC for all parameters ranged from 0.313 to 0.975 (Table 2). For gait parameters including both feet, the ICC ranged from 0.663 to 0.990. More specifically, ICC for cadence was excellent (ICC = 0.990, *p* = 0.000), for double contact was good (ICC = 0.784, *p* = 0.000), for walking speed was excellent (ICC = 0.916, *p* = 0.000) and for symmetry was moderate (ICC = 0.663, *p* = 0.060).

As regards the ICC in gait parameters of left foot, circumduction was excellent (ICC = 0.949, *p* = 0.000), clearance was moderate (ICC = 0.579, *p* = 0.108), contact time was moderate (ICC = 0.575, *p* = 0.036), flat foot time was moderate (ICC = 0.657, *p* = 0.057), foot progression angle was good (ICC = 0.755, *p* = 0.014), heel strike angle was good (ICC = 0.792, *p* = 0.009), loading time was excellent (ICC = 0.918, *p* = 0.000), propulsion rate was poor (ICC = 0.476, *p* = 0.065), propulsion time was moderate (ICC= 0.546, *p* = 0.038), stride duration was excellent (ICC = 0.975, *p* = 0.000), stride length was excellent (ICC = 0.912, *p* = 0.000), supination angle during heel off was moderate (ICC = 0.730, *p* = 0.028), supination angle during heel strike was good (ICC = 0.710, *p* = 0.032), supination angle during toe off was poor (ICC = 0.374, *p* = 0.239), supination angle during toe-strike was poor (ICC = 0.350, *p* = 0.24), while swing time was moderate (ICC = 0.663, *p* = 0.060).

Concerning ICC in gait parameters of the right foot, circumduction was moderate (ICC = 0.723, *p* = 0.017), clearance was poor (ICC = 0.337, *p* = 0.247), contact time was moderate (ICC = 0.568, *p* = 0.026), flat foot time was moderate (ICC = 0.577, *p* = 0.106), foot progression angle was good (ICC = 0.782, *p* = 0.016), heel strike angle was moderate (ICC = 0.706, *p* = 0.009), loading time was excellent (ICC = 0.862, *p* = 0.001), propulsion rate was moderate (ICC = 0.563, *p* = 0.092), propulsion time was moderate (ICC = 0.699, *p* = 0.039), stride duration was excellent (ICC = 0.962, *p* = 0.000), stride length was excellent (ICC = 0.939, *p* = 0.000), supination angle during heel off was moderate (ICC = 0.710, *p* = 0.032), supination angle during heel strike was moderate (ICC = 0.576, *p* = 0.108), supination angle during toe off was poor (ICC = 0.313, *p* = 0.202), supination angle during the toe strike was moderate (ICC = 0.540, *p* = 0.126) and swing time was also moderate (ICC = 0.525, *p* = 0.047).

## 4. Discussion

This paper introduced the PODOSmart^®^ insole, a system containing wireless sensors capable of measuring gait parameters. PODOSmart^®^ insoles are flexible and may be inserted into the participant’s shoes; therefore, it is less likely to affect the user’s natural gait.

The validation of portable gait analysis systems is becoming an important study due to the increasing availability of these technologies for the analysis of the asymptomatic or pathological gait. Thus, we examined the accuracy of the PODOSmart^®^, which is a portable inertial measurement unit (IMU) system compared to the Vicon gold standard optoelectronic system. Measurements via PODOSmart^®^ and Vicon concerning walking trials were performed simultaneously for a total of 11 subjects.

PODOSmart^®^ measurements were valid and accurate compared to the gold standard Vicon system, with acceptable ICC values. The validation results indicate a high matching between most of the spatiotemporal gait parameters computed by PODOSmart^®^ insoles and Vicon motion capturing system. More specifically, in temporal gait parameters such as cadence, walking speed, and step duration, ICC values were greater than 0.916. Concerning spatial gait parameters, such as stride length, clearance, and circumduction, ICC values ranged from 0.337 (clearance in cm for the right foot) to 0.949 (circumduction in cm for the left foot). Regarding joint angle parameters in gait, ICC values varied from 0.350 (for supination angle at toe strike time) to 0.792 (for heel strike angle).

This paper is the first study examining 20 gait variables provided by PODOSmart^®^ insoles and validated using Vicon human motion capturing technology. In the recent studies related to the validation of insole devices for gait analysis, previous efforts examined the validity for only a few variables such as step counting, cadence [24,25], or a small number of spatiotemporal gait characteristics [2,17]. Consequently, due to methodological differences, direct comparisons are not possible. Aiming to test the validity of sensor-based insoles for gait analysis, different methodological approaches are presented in recent bibliography. Force platforms [2,15,19,24], plantar pressure test plates [22], electronic walkways with built-in pressure sensors [21], instrumented walkways [23], the FDM-S System (Zebris) [17] and a stereophotogrammetry-based system [20] (Vicon) have been used for the validation of gait analysis insoles, depending on the type of examined variables. Thus, our findings are in line with other similar studies where the objective was to validate low-cost, portable, and wireless tools used for gait analysis [17,24]. The study of Burns et al. (2019) reported ICC = 0.890 for both feet in ground reaction force and ICC ranging from 0.95 to 0.96 for both feet in contact time and impulse [24]. In addition, Braun et al. (2015), in their study, reported that intraclass correlation for the validation of a new fully integrated gait analysis insole was >0.796 for spatiotemporal and kinematic gait parameters [17]. In compliance with the present findings, the study of Macleod et al. (2013), used Vicon system in order to test the validity of wi-GAT and reported strong agreement in stride length and duration, cadence, stance duration and walking speed (ICC of 0.94–0.996) and less agreement in gait parameters such as percentage of stance phase, percentage of swing phase and double support time (ICC of 0.299–0.847) between the two systems [20].

Furthermore, the methodology followed for this study required only short acquisitions, due to the fact that Vicon system capturing volume was set to approximately 6 m × 6 m × 3 m. Although PODOSmart^®^ insoles are designed for long-lasting recordings, the present findings indicate that PODOSmart^®^ insoles algorithms allow the use of PODOSmart^®^ insoles in short-term recordings. Gait analysis with foot mounted IMU sensors is well defined in the literature [13,28,29,30]. Sensor orientation, related to the global reference, can be computed with complementary or Kalman filters [28]. These filters allow to compute foot angle relative to the ground without drift. In addition, spatial parameters, including linear velocity or displacement can be estimated with low computational cost using strap-down integration methods with stride-to-stride integration [29] or more complex Kalman filter with zero velocity update (ZUPT) input [30]. Additionally, gait events such as heel strike or toe off may be computed using technics such as rule-based methods, machine learning or wavelet transform [13]. Based on these events, temporal parameters such as contact time, swing time or cadence can be calculated with advanced accuracy and precision.

This study has some limitations that should be acknowledged. Firstly, because of some Vicon noisy data, the annotation of some key timestamps (toe strike, toe off) sometimes was not accurate enough. This caused errors in parameter computation, in particular for the ankle joint parameters (e.g., supination angles) whose values are very sensible to timestamp. Secondly, previous published efforts regarding validation of the gait analysis insoles were performed using samples ranging from one [37] to forty [38] participants, with most of them using samples with around ten participants. The present study sample size was relatively small, which could limit the accuracy of the results. In addition, one point to consider is that only a few steps were captured for each participant. In such a validation process, the Vicon motion capture system uses very few steps; therefore, only two gait cycles were used for analysis because of the exclusion of the acceleration and deceleration steps. Thus, when using the PODOSmart^®^ insoles, only a part of captured data can be used for analyses in order to compare the same number of steps and strides. In that context, this kind of process may not reflect the natural variability of human gait. However, the advantage of ease of use and the less expensive use, without the technical education of an IMU insole device, outweighs the disadvantage of slightly reduced data accuracy, mainly in variables concerning stance phase, as observed from the results of this study.

Further studies need to be performed, including more subjects and/or more steps using external video capturing of the acquisitions to further validate PODOSmart^®^ system. It will be useful to use pressure sensors to detect the gait cycle’s events more accurately. Such results could also be used for the validation of different activities (e.g., running) and for populations presenting pathological gait (orthopedic and neurologic patients).

## 5. Conclusions

The IMU-based PODOSmart^®^ insole device provides valid measurements of the parameters of normal walking compared to the gold standard Vicon. Further investigations are in progress to increase the study confidence by increasing the number of subjects, studied parameters and the number of analyzed steps. This future validation will also include the use of PODOSmart^®^ insoles measuring gait parameters of more specified age groups and female subjects as age and gender significantly affects human gait. Additionally, further investigation of the validity of PODOSmart^®^ is required for different activities (e.g., running) and pathological gait in neurologic or orthopedic patients, since gait patterns of pathological population are quite different compared to healthy individuals.

## Figures and Tables

**Table 1:** Gait variables provided by PODOSmart^®^.

Walking Profile	Oscillating Phase	Stance Phase
Contact time (ms)	Foot progression angle (°)	Heel strike (°)
Flying time (ms)	Clearance (in centimetres)	Toe strike (°)
Taligrade (ms)	Steppage (°)	Heel off (°)
Plantigrade (ms)	Walking speed (in km per hour)	Toe off (°)
Digitigrade (ms)	Stride length (in meters)	
	Cadence (in steps per minute)	

**Table 2:** Descriptive statistics and ICC values from Vicon and PODOSmart^®^ insoles data of each variable.

	Side	Method	Means and S.D.	Reliability	ICC	Sig.
Cadence (setps/min)	-	Vicon	66.73 ± 21.13	Excellent	0.990	0.000
		PODOSmart^®^	67.68 ± 22.72			
Circumduction (cm)	L	Vicon	2.16 ± 1.00	Excellent	0.949	0.000
		PODOSmart^®^	2.09 ± 1.05			
	R	Vicon	3.54 ± 0.97	Moderate	0.723	0.017
		PODOSmart^®^	4.10 ± 1.32			
Clearance (cm)	L	Vicon	1.46 ± 0.49	Moderate	0.579	0.108
		PODOSmart^®^	1.50 ± 0.57			
	R	Vicon	1.04 ± 1.11	Poor	0.337	0.247
		PODOSmart^®^	2.03 ± 2.30			
Contact time (%)	L	Vicon	63.00 ± 2.72	Moderate	0.575	0.036
		PODOSmart^®^	61.18 ± 1.25			
	R	Vicon	62.27 ± 1.19	Moderate	0.568	0.026
		PODOSmart^®^	60.82 ± 1.72			
Double contact (%)	-	Vicon	12.09 ± 1.11	Good	0.784	0.000
		PODOSmart^®^	11.18 ± 1.08			
Flat foot time (ms)	L	Vicon	370.00 ± 50.40	Moderate	0.657	0.057
		PODOSmart^®^	380.91 ± 38.44			
	R	Vicon	391.82 ± 121.06	Moderate	0.577	0.106
		PODOSmart^®^	410.45 ± 100.94			
Foot progression angle (°)	L	Vicon	6.75 ± 4.51	Good	0.755	0.014
		PODOSmart^®^	9.45 ± 8.86			
	R	Vicon	9.00 ± 4.59	Good	0.782	0.016
		PODOSmart^®^	9.28 ± 3.54			
Heel strike angle (°)	L	Vicon	18.20 ± 5.69	Good	0.792	0.009
		PODOSmart^®^	16.48 ± 5.03			
	R	Vicon	17.60 ± 5.40	Moderate	0.706	0.032
		PODOSmart^®^	15.89 ± 5.17			
Loading time (ms)	L	Vicon	221.82 ± 93.37	Excellent	0.918	0.000
		PODOSmart^®^	195.27 ± 74.70			
	R	Vicon	186.36 ± 72.43	Good	0.862	0.001
		PODOSmart^®^	156.45 ± 76.43			
Propulsion rate (%)	L	Vicon	37.45 ± 7.19	Poor	0.476	0.065
		PODOSmart^®^	29.82 ± 7.65			
	R	Vicon	36.91 ± 14.84	Moderate	0.563	0.092
		PODOSmart^®^	31.64 ± 7.38			
Propulsion time (ms)	L	Vicon	232.73 ± 44.74	Moderate	0.546	0.038
		PODOSmart^®^	265.55 ± 19.06			
	R	Vicon	258.64 ± 56.40	Moderate	0.699	0.039
		PODOSmart^®^	268.73 ± 31.90			
Walking speed (km/h)	-	Vicon	2.95 ± 0.37	Excellent	0.916	0.000
		PODOSmart^®^	2.92 ± 0.36			
Stride duration (ms)	L	Vicon	1.357.27 ± 100.8	Excellent	0.975	0.000
		PODOSmart^®^	1.349.36 ± 104.6			
	R	Vicon	1.382.73 ± 101.0	Excellent	0.962	0.000
		PODOSmart^®^	1.383.82 ± 116.1			
Stride length (m)	L	Vicon	1.12 ± 0.08	Excellent	0.912	0.000
		PODOSmart^®^	1.10 ± 0.09			
	R	Vicon	1.14 ± 0.09	Excellent	0.939	0.000
		PODOSmart^®^	1.15 ± 0.11			
Supination angle HO (°)	L	Vicon	−5.96 ± 3.88	Moderate	0.730	0.028
		PODOSmart^®^	−6.55 ± 1.63			
	R	Vicon	−6.92 ± 4.34	Moderate	0.710	0.032
		PODOSmart^®^	−7.94 ± 2.89			
Supination angle HS (°)	L	Vicon	−16.84 ± 4.37	Poor	0.438	0.139
		PODOSmart^®^	−14.35 ± 1.86			
	R	Vicon	−15.10 ± 9.78	Moderate	0.576	0.108
		PODOSmart^®^	−16.15 ± 5.04			
Supination angle TO (°)	L	Vicon	−9.35 ± 7.11	Poor	0.374	0.239
		PODOSmart^®^	−7.35 ± 4.06			
	R	Vicon	−11.59 ± 6.31	Poor	0.313	0.202
		PODOSmart^®^	−6.55 ± 3.30			
Supination angle TS (°)	L	Vicon	−8.98 ± 3.89	Poor	0.350	0.244
		PODOSmart^®^	−7.48 ± 2.10			
	R	Vicon	−8.78 ± 3.96	Moderate	0.540	0.126
		PODOSmart^®^	−7.95 ± 3.33			
Swing time (%)	L	Vicon	37.27 ± 2.45	Moderate	0.577	0.043
		PODOSmart^®^	38.82 ± 1.25			
	R	Vicon	37.73 ± 1.27	Moderate	0.525	0.047
		PODOSmart^®^	39.18 ± 1.72			
Symmetry (%)	-	Vicon	92.64 ± 2.50	Moderate	0.663	0.060
		PODOSmart^®^	93.00 ± 4.26			

## Data Availability

The data presented in this study are available on request from the corresponding author. The data are not publicly available due to privacy and ethical restrictions.
